# Evidence that Lokern virus (family *Peribunyaviridae*) is a reassortant that acquired its small and large genome segments from Main Drain virus and its medium genome segment from an undiscovered virus

**DOI:** 10.1186/s12985-018-1031-6

**Published:** 2018-08-06

**Authors:** Chandra S. Tangudu, Jermilia Charles, Bradley J. Blitvich

**Affiliations:** 1 Department of Veterinary Microbiology and Preventive Medicine, College of Veterinary Medicine, Iowa State University, Ames, IA USA; 2 2116 Veterinary Medicine, Iowa State University, Ames, Iowa 50011 USA

**Keywords:** Lokern virus, Main drain virus, *Orthobunyavirus*, *Peribunyaviridae*, Reassortant, Genome sequence, High-throughput sequencing

## Abstract

**Background:**

Lokern virus (LOKV) is a poorly characterized arthropod-borne virus belonging to the genus *Orthobunyavirus* (family *Peribunyaviridae*). All viruses in this genus have tripartite, single-stranded, negative-sense RNA genomes, and the three RNA segments are designated as small, (S), medium (M) and large (L). A 559 nt. region of the M RNA segment of LOKV has been sequenced and there are no sequence data available for its S or L RNA segments. The purpose of this study was to sequence the genome of LOKV.

**Methods:**

The genome of LOKV was fully sequenced by unbiased high-throughput sequencing, 5′ and 3′ rapid amplification of cDNA ends, reverse transcription-polymerase chain reaction and Sanger sequencing.

**Results:**

The S and L RNA segments of LOKV consist of 952 and 6864 nt. respectively and both have 99.0% nucleotide identity with the corresponding regions of Main Drain virus (MDV). In contrast, the 4450-nt. M RNA segment has only 59.0% nucleotide identity with the corresponding region of MDV and no more than 72.7% nucleotide identity with all other M RNA segment sequences in the Genbank database. Phylogenetic data support these findings.

**Conclusions:**

This study provides evidence that LOKV is a natural reassortant that acquired its S and L RNA segments from MDV and its M RNA segment from an undiscovered, and possibly extinct, virus. The availability of complete genome sequence data facilitates the accurate detection, identification and diagnosis of viruses and viral infections, and this is especially true for viruses with segmented genomes because it can be difficult or even impossible to differentiate between reassortants and their precursors when incomplete sequence data are available.

## Background

Lokern virus (LOKV) is a poorly characterized virus in the genus *Orthobunyavirus* (family *Peribunyaviridae*). The genus has previously been separated into serogroups, with LOKV classified in the Bunyamwera (BUN) serogroup [1]. Other viruses in this serogroup include Batai virus (BATV), Bunyamwera virus (BUNV), Cache Valley virus (CVV), Maguari virus (MAGV), Ngari virus (NRIV), Northway virus (NORV), and Tensaw virus (TENV). LOKV was originally isolated from *Culex tarsalis* mosquitoes in California in 1962 [2]. Additional isolations have been made from arthropods of various species as well as rabbits and hares in the western half of the United States, with most isolates recovered from *Culicoides* spp. midges [2–5]. Serologic data indicate that the geographic range of LOKV also includes the eastern half of the United States [6]. LOKV is not a recognized pathogen of humans or vertebrate animals but its ability to cause disease has not been widely investigated.

All viruses in the genus *Orthobunyavirus* possess a tripartite, single-stranded, negative-sense RNA genome [7]. The three RNA segments are designated as small, (S), medium (M) and large (L) and are approximately 1.0, 4.5 and 6.9 kb in length, respectively. Complementary and highly conserved nucleotide sequences are located at the 5′ and 3′ termini of each RNA segment. Base pairing of these sequences results in the formation of panhandle structures assumed to be required for the initiation of genome transcription and replication [8, 9]. The S RNA segment codes the nucleocapsid (N) protein and a nonstructural protein NS_S_ in overlapping reading frames [10]. The M RNA segment codes for a polyprotein that is post-translationally processed to generate the N- and C-terminal glycoproteins, Gn and Gc, and a nonstructural protein NS_M_ [11]. The L RNA segment codes for the L protein which functions as the RNA-dependent RNA polymerase [12].

The exchange of RNA segments (genome reassortment) between closely-related orthobunyaviruses can occur when a vector or host is co-infected with two or more viruses [13, 14]. Reassortment is considered to be a major means of evolution for these viruses because the resulting progeny can possess new and advantageous genetic and phenotypic traits. Reassortment sometimes generates viruses that are more pathogenic than their donor viruses. One example is NRIV which acquired its S and L RNA segments from BUNV and its M RNA segment from BATV [15, 16]. NRIV has been associated with large outbreaks of hemorrhagic fever in humans in East Africa while the most serious disease manifestations associated with its precursor viruses are low-grade fever and myalgia.

The genome of LOKV has not been fully sequenced. A 559 nt. region of the M RNA segment that spans part of the 5′ untranslated region (UTR) and Gn coding region has been sequenced (Genbank Accession no. AY593736.1) and no sequence data are available for the S or L RNA segments. The objective of this study is to fully sequence the genome of LOKV and to determine its genetic and phylogenetic relationships with other BUN serogroup viruses.

## Methods

### Virus

LOKV (isolate FMS 4332) was obtained from the World Reference Center for Emerging Viruses and Arboviruses at the University of Texas Medical Branch in Galveston, TX. The virus had been passaged 13 times in suckling mouse brains prior to receipt and underwent an additional passage in African Green Monkey kidney (Vero) cells at Iowa State University.

### High-throughput sequencing

Total RNA was extracted from subconfluent monolayers of LOKV-infected Vero cells using Trizol Reagent (ThermoFisher, Carlsbad, CA), fragmented using RNase III (New England BioLabs, Ipswich, MA) and assessed for quality using an Agilent 2100 Bioanalyzer (Agilent, Santa Clara, CA). Libraries were constructed using the Ion Total RNA-Seq Kit v2 (ThermoFisher) and assessed for quality and analyzed at the Genomic Technologies Facility at Iowa State University using an Ion Proton Sequencer (ThermoFisher). Ion-Torrent reads were mapped to the genome of *Chlorocebus sabaeus* using Bowtie 2 in order to identify host cell sequences [17]. Unmapped reads were analyzed using the sortMeRNA program to remove rRNA-related reads [18]. All remaining reads with Phred values ≥ 33 were subjected to de novo SPAdes (ver 3.5.0) assembly [19]. Contigs were mapped to a reference orthobunyavirus genome (MDV for S and L RNA segment sequences; CVV for M RNA segment sequences) using LASTZ [20]. Alignment files were manually verified on Tablet [21].

### Reverse transcription-polymerase chain reaction and sanger sequencing

Reverse transcription-polymerase chain reaction (RT-PCR) and Sanger sequencing were performed to verify LOKV sequences generated by high-throughput sequencing and to close gaps between contigs. Complementary DNAs were generated using Superscript III reverse transcriptase (ThermoFisher) and PCRs were performed using high-fidelity *Taq* polymerase (ThermoFisher) in accordance to the manufacturer’s instructions. Primers were designed from the sequences generated by high-throughput sequencing and are available upon request. RT-PCR products were purified using the purelink gel extraction kit (ThermoFisher) and sequenced using a 3730 × 1 DNA sequencer (Applied Biosystems, Foster City, CA).

### 5′ and 3′ rapid amplification of cDNA ends

The 5′ and 3′ ends of each RNA segment were identified using 5′ and 3′ rapid amplification of cDNA ends, respectively. Briefly, 20 pmol of pre-adenylated DNA adaptor (5′-rApp/TGGAATTCTCGGGTGCCAAGGT/ddC-3′) was ligated to 1 μg of viral genomic and anti-genomic RNAs using 200 U of T4 RNA ligase 2 (New England BioLabs) and incubated for 1 h at 25 °C. Ligated products were recovered by ethanol precipitation and complementary cDNAs were created using Superscript III reverse transcriptase and an adapter-specific primer. PCRs were performed using adapter- and gene-specific primers, and RT-PCR products were purified and subjected to Sanger sequencing.

### Nucleotide and amino acid sequence alignments

Nucleotide and predicted amino acid sequences of LOKV were aligned with other sequences in the Genbank database by application of BLASTn and BLASTp, respectively. Alignments were also performed using Clustal Omega (available at http://www.ebi.ac.uk/Tools/msa/clustalo/) in order to calculate percent nucleotide and amino acid identities between select sequences.

### Phylogenetic analysis

Nucleotide sequences were alignment using the program Multiple Alignment Using Fast Fourier Transform. A Markov Chain Monte Carlo-based Bayesian analysis was performed by MrBayes using the general time reversible plus invariant sites plus gamma distribution 4 model [22]. To reach congruence, 10 million iterations were run and sampled every 1000th iteration. The maximum clade credibility tree was created using TreeAnnotator (version 1.8.2) with 10% burn-in [23] and visualized with FigTree (version 1.4.0).

## Results

The complete nucleotide sequences of the S, M and L RNA segments of LOKV were determined (Genbank Accession nos. MG696865, MG820264 and MG828823, respectively). The S RNA segment consists of 952 nt., and is most closely related to the corresponding region of MDV with 99.0% nt. identity, followed by NRIV and NORV with 85.0 and 84.6% nt. identity, respectively (Table 1). The S RNA segment of LOKV contains two open reading frames (ORFs). The most 5’ AUG initiates the longest ORF which encompasses nucleotide positions 77 to 778, and encodes for the N protein. The predicted translation product consists of 233 amino acids, and is most closely related to the N protein of MDV with 99.6% identity. The second ORF encompasses nucleotide positions 96 to 401 and encodes for the NS_S_ protein. The predicted translation product consists of 101 amino acids and is identical to the NS_S_ protein of MDV.

**Table 1:** Genetic relatedness of the small, medium and large genome segments of Lokern virus and its closest known relatives

Virus	Lokern virus						
	% nucleotide identity			% amino acid identity			
	Small	Medium	Large	N	NS_S_	^a^M	L
Abbey Lake virus	70.9	59.8	71.4	73.4	64.0	55.0	76.5
Batai virus	79.5	68.7	73.1	88.4	90.1	71.5	80.7
Birao virus	78.3	^b^–	–	87.6	85.0	–	–
Bozo virus	76.3	–	–	79.0	78.2	–	–
Bunyamwera virus	80.0	63.4	73.5	89.7	86.2	62.4	81.1
Cache Valley virus	83.5	72.5	76.1	89.3	94.1	78.4	85.5
Cholul virus	83.3	58.6	–	89.3	94.1	54.4	–
Fort Sherman virus	81.8	72.7	76.3	91.0	93.1	77.9	86.0
Germiston virus	71.1	59.3	–	73.8	67.0	54.7	–
Ilesha virus	79.6	67.4	73.4	86.7	85.2	67.0	80.2
Main Drain virus	99.0	59.0	99.0	99.6	100.0	53.9	99.0
Maguari virus	83.0	72.2	76.2	91.4	93.1	77.6	86.6
Mboke virus	78.3	–	–	86.3	84.2	–	–
Ngari virus	85.0	68.6	74.0	89.7	86.2	71.0	81.1
Northway virus	84.6	71.4	76.8	91.4	95.0	76.9	86.7
Playas virus	83.3	72.4	76.1	89.3	94.1	78.0	85.6
Potosi virus	83.2	59.1	75.5	89.3	94.1	54.4	84.8
Shokwe virus	81.0	–	–	90.1	88.1	–	–
Tensaw virus	82.4	70.4	76.5	91.4	88.1	74.8	86.1
Tlacotalpan virus	82.7	72.7	76.1	89.3	93.1	78.4	85.7
Xingu virus	78.8	–	–	86.3	84.2	–	–

^a^Alignments were performed using the Gn-NS_M_-Gc polyprotein; ^b^Complete sequence data not available; Genbank Accession Nos. used for the nucleotide sequence alignments are as follows: Abbey Lake virus (KJ710424.1, KJ710423.1, KJ710425.1), Bunyamwera virus (NC_001927.1, M11852.1, X14383.1), Batai virus (KU746869.1, KU746870.1, KU746871.1), Birao virus (AM711131.1), Bozo virus (AM711132.1), Cache Valley virus (KX100133.1, KX100134.1, KX100135.1), Cholul virus (EU879062.3, JN808310.1), Fort Sherman virus (KX100130.1, KX100131.1, KX100132.1), Germiston virus (M19420.1, M21951.1), Ilesha virus (KF234073.1, KF234074.1, KF234075.1), Main Drain virus (X73469.1, EU004187.1, MG652604), Maguari virus (KY910431.1, KY910430.1, KY910429.1), Mboke virus (AY593727.1), Ngari virus (KM507341.1, KM514677.1, KM507334.1), Northway virus (X73470.1, EU004188.1, MG544835), Playas virus (KX100121.1, KX100122.1, KX100123.1), Potosi virus (MF066370.1, MF066369.1, MF066368.1), Shokwe virus (EU564831.1), Tensaw virus (FJ943505.1, FJ943506.1, FJ943509.1), Tlacotalpan virus (KX100118.1, KX100119.1, KX100120.1) and Xingu virus (EU564830.1). Genbank Accession Nos. used for amino acid sequence alignments can be accessed by following the links in the Genbank entries listed above

The M RNA segment of LOKV consists of 4450 nt., and is most closely related to the corresponding regions of CVV and its variants, Fort Sherman, Playas and Tlacotalpan viruses, with 72.4 to 72.7% nt. identity, followed by MAGV and NORV with 72.2 and 71.4% nt. identity, respectively (Table 1). The partial M RNA segment sequence of LOKV previously available in the Genbank database has 99.6% nt. identity with the corresponding region of our sequence. The sequences differ in two nucleotide positions; there is a nucleotide substitution in the 5’ UTR and a synonymous substitution in the Gn coding region. Pairwise nucleotide sequence alignments were also performed using the partial M RNA segment sequences of Birao, Bozo, Mboke, Shokwe, and Xingu viruses, revealing 60.2 to 68.8% nt. identity with the corresponding region of LOKV. The M RNA segment of LOKV contains a single ORF that encodes for a polyprotein of 1434 amino acids that is predicted to yield Gn, Gc and NS_M_ proteins of 286, 866 and 173 amino acids, respectively. The deduced amino acid sequence of the polyprotein has greatest identity (77.9 to 78.4%) with the corresponding regions of CVV and its variants (Table 1). Alignments were also performed using the deduced amino acid sequences of the individual proteins, revealing that the Gn, Gc and NS_M_ proteins of LOKV have at least 87.0, 79.1 and 67.0% identity, respectively to the corresponding proteins of CVV and its variants.

The L RNA segment of LOKV consists of 6864 nt., and is most closely related to the corresponding region of MDV with 99.0% nt. identity, followed by NORV and TENV with 86.7 and 86.1% nt. identity, respectively (Table 1). The L RNA segment of LOKV contains a single ORF that encompasses nucleotide positions 49 to 6765 and encodes for the L protein. The predicted translation product consists of 1434 residues and has 99.0% identity with the L protein of MDV.

**Fig. 1:**
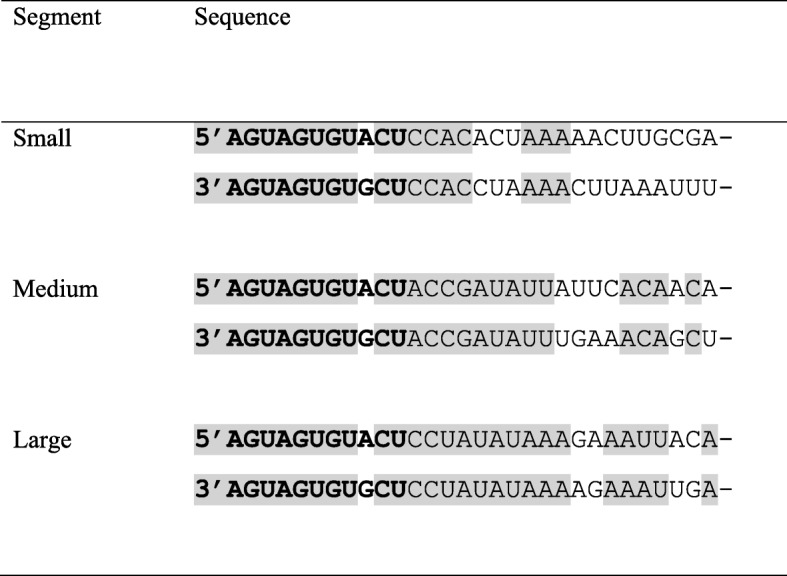
Terminal nucleotides at the 5′ and 3′ ends of each RNA segment of Lokern virus

Complementary sequences were identified at the 5′ and 3′ ends of all three RNA segments of LOKV (Fig. 1). Seventeen of the 21 terminal nucleotides at the 5′ and 3′ ends of the S RNA segment are complementary, and an inspection of the M and L RNA segment sequences revealed that 19 of 20 and 24 of 27 nt., respectively are complementary. The first 11 nt. at the 5′ ends of all three segments are characterized by the sequence 5’-AGUAGUGUACU-3′. The same sequence is present at the 3′ ends of all three segments, when in reverse complementary orientation, aside from a mismatch at positions 9 and − 9.

**Fig. 2:**
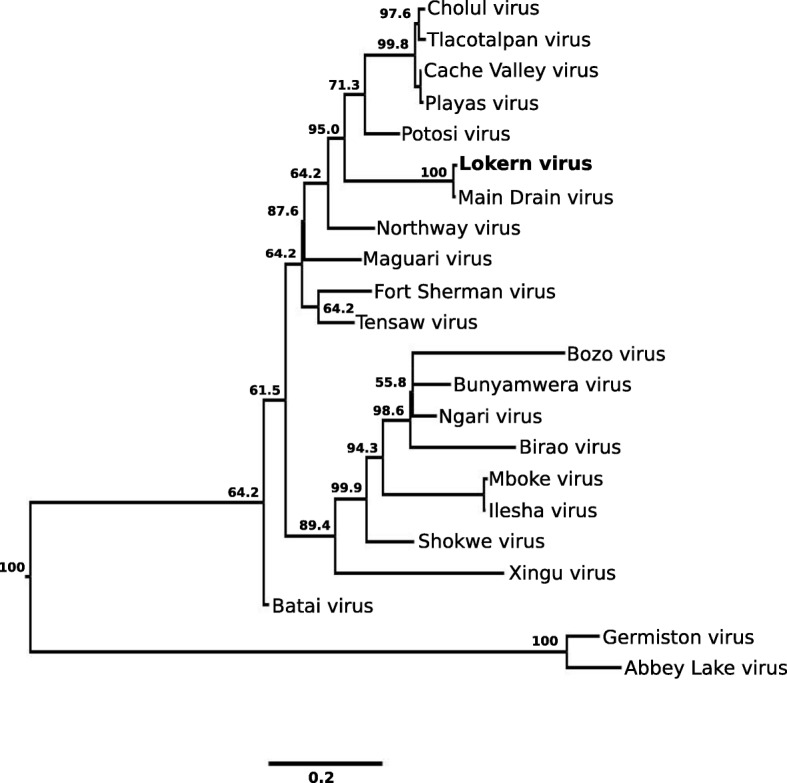
Phylogenetic analysis of the small RNA segment of Lokern virus. The analysis is based on a 702-nt. region of the small RNA segments of Lokern virus and the corresponding regions of all other Bunyamwera serogroup viruses for which these sequence data are available. Select posterior probabilities are denoted as percentages under nodes. Branch lengths are proportional to number of nucleotide differences. Genbank Accession numbers used in the analysis are listed in the footnote of Table 1

**Fig. 3:**
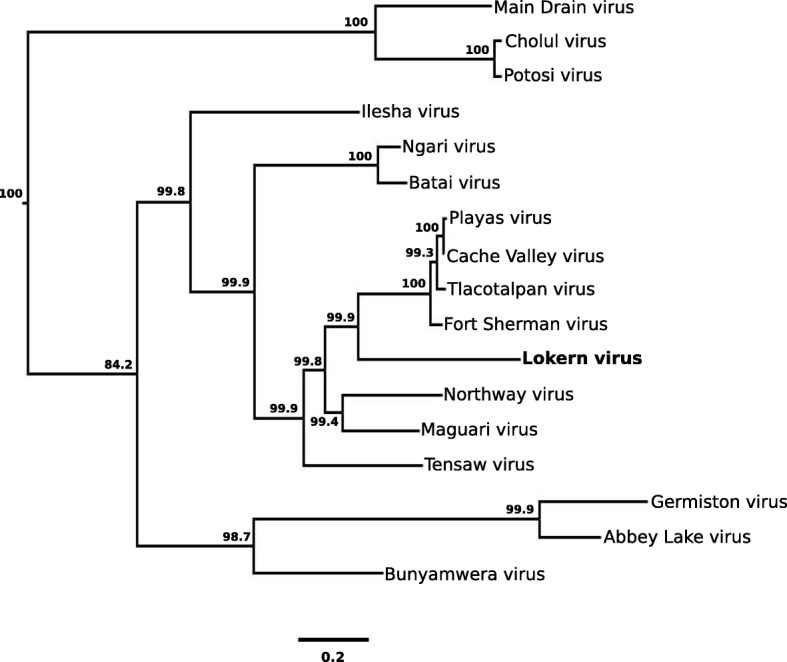
Phylogenetic analysis of the medium RNA segment of Lokern virus. The analysis is based on a 4345-nt. region of the medium RNA segment of Lokern virus and the corresponding regions of all other Bunyamwera serogroup viruses for which these sequence data are available. Select posterior probabilities are denoted as percentages under nodes. Branch lengths are proportional to number of nucleotide differences. Genbank Accession numbers are listed in the footnote of Table 1

**Fig. 4:**
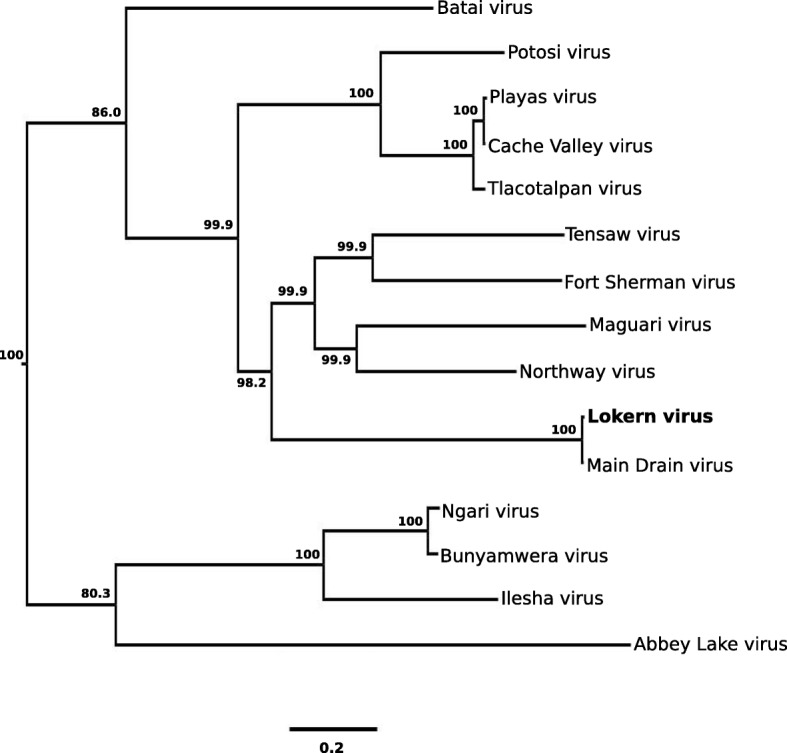
Phylogenetic analysis of the large RNA segment of Lokern virus. The analysis is based on a 6722-nt. region of the large RNA segment of Lokern virus and the corresponding regions of all other Bunyamwera serogroup viruses for which these sequence data are available. Select posterior probabilities are denoted as percentages under nodes. Branch lengths are proportional to number of nucleotide differences. Genbank Accession numbers are listed in the footnote of Table 1

Phylogenetic trees were constructed with Bayesian methods using the nucleotide sequences of the S, M and L RNA segments of LOKV and selected other orthobunyaviruses (Figs. 2, 3, 4). The S RNA segment sequence of LOKV is most closely related phylogenetically to the corresponding segment of MDV. The Bayesian posterior support for this topological arrangement is 100%. Likewise, in the Bayesian tree constructed using L RNA segment sequences, LOKV is most closely related to MDV with strong posterior support for this grouping (100%). In contrast, the M RNA segment sequence of LOKV is most closely related phylogenetically to the corresponding regions of CVV and its variants. The posterior support for this topological arrangement is 99.9%. These viruses, along with NORV, MAGV, and TENV, comprise a distinct clade for which there is strong posterior support (99.9%).

## Discussion

This study provides evidence that LOKV is a natural reassortant that acquired its S and L RNA segments from MDV and its M RNA segment from an undiscovered, and possibly extinct, virus. MDV was assumed to be one of the donor viruses because its S and L RNA segments are almost identical to those of LOKV. The two viruses have similar geographic distributions, and vector and host associations, thus providing the opportunity for reassortment to occur. LOKV and MDV both occur in the western half of the United States, and most arthropod- and vertebrate-derived isolations of each virus have been from *Culicoides* spp. midges and rabbits, respectively [3]. Another, albeit less likely, explanation is that LOKV and MDV are both reassortants that acquired their S and L RNA segments from the same ancestor(s). It has been suggested that MDV is a reassortant that acquired its S RNA segment from MAGV or NORV, its M RNA segment from Potosi or Kairi viruses, and its L RNA segment from CVV or MAGV [24].

The M RNA segment of LOKV was assumed to be acquired from an undiscovered virus because it has no more than 72.7% nt. identity to the M RNA segments of all other known orthobunyaviruses. Another, less likely, explanation is that the M segment donor is a known virus but the reassortant event was not recent, thus providing sufficient time for a considerable amount of sequence divergence to occur by genetic drift. Of all viruses positioned basally to LOKV in Fig. 3, MAGV and NORV are its closest relatives. It is unlikely that MAGV is the M RNA segment donor because its known geographic distribution does not overlap with LOKV. MAGV occurs in Latin American and the Caribbean, but has never been detected in the United States (Centers for Disease Control and Prevention Arbovirus Catalog, https://wwwn.cdc.gov/arbocat/VirusDetails.aspx?ID=276&SID=12, accessed April 2, 2018). However, NORV and LOKV have overlapping geographic distributions and vector and host ranges. NORV occurs in the western half of the United States and Canada, and has been isolated from rabbits and other rodents as well as mosquitoes [3, 25].

Our data support earlier studies which have shown that the most common reassortment event among BUN serogroup viruses is the M RNA segment exchange. In a classic study, heterologous crosses were performed in the laboratory using BUNV/BATV and BUNV/MAGV, with all resulting recombinants acquiring their S and L RNA segments from the same parental virus [26]. Furthermore, MDV, NRIV and Potosi virus are all natural reassortants that arose from M RNA segment exchanges [15, 16, 24, 27]. Cholul virus is an exception; it is a natural reassortant that arose from an S RNA segment exchange [28]. The M RNA segment exchange is not the preferred reassortment pattern for California serogroup viruses which usually have homologous M-L and S-M associations and Group C viruses which appear to favor homologous M-L associations [29, 30].

A characteristic feature of the genomes of all viruses in the genus *Orthobunyavirus* is the presence of complementary nucleotide sequences at the 5′ and 3′ termini of each RNA segment [7, 31–33]. The sequences are highly conserved between each RNA segment of a virus and between each virus in the genus. The consensus sequence consists of 11 nt. and is defined as 5’-AGUAGUGUACU-3′, with a mismatch at positions 9 and − 9 at each end, as we observed with LOKV. The terminal 11 nt. are followed by 3 to 4 more nucleotides that are complementary and highly conserved on a segment-specific basis, followed by several more nucleotides that are complementary and usually unique to the individual virus and segment [29, 30]. An inspection of the genome sequence of LOKV revealed the presence of terminal complementary sequences of 21 to 27 nt., with one to four mismatches, at the terminal ends of each segment, consistent with earlier reports that the region of complementary extends beyond the highly conserved 11 nt. consensus sequence.

## Conclusions

To conclude, the sequence and phylogenetic data presented in this study provide evidence that LOKV is a reassortant that arose from an M RNA segment exchange. We consider it most likely that LOKV acquired its S and L RNA segments from MDV and its M RNA segment from an unrecognized virus, but additional explanations are also provided. Complete genome sequence data is needed for the accurate detection and identification of viruses, particularly when the virus in question has a segmented genome because reassortants and their precursors could otherwise be mistaken for one another.

## Abbreviations

BATV: Batai virus
BUN: Bunyamwera
BUNV: Bunyamwera virus
CVV: Cache Valley virus
L: Large
LOKV: Lokern virus
M: Medium
MAGV: Maguari virus
N: Nucleocapsid
NORV: Northway virus
NRIV: Ngari virus
ORF: Open reading frame
RT-PCR: Reverse transcription-polymerase chain reaction
S: Small
TENV: Tensaw virus
UTR: Untranslated region
